# Traditional and Non-Traditional Grain-Based Products: Addressing Challenges Through Formulation and Processing

**DOI:** 10.3390/foods15101700

**Published:** 2026-05-12

**Authors:** Andrea Bresciani, Catrin E. Tyl, Alessandra Marti

**Affiliations:** 1 Department of Food, Environmental and Nutritional Sciences (DeFENS), Università degli Studi di Milano, Via G. Celoria 2, 20133 Milan, Italy; 2 Faculty of Chemistry, Biotechnology and Food Science, Norwegian University of Life Science, 1433 Ås, Norway

Grain-based foods have been integral components of the diets of people around the world for millennia. Yet, there is growing consumer demand for products with novel nutritional, sensory, and technological characteristics [1,2]. The papers collected in this Special Issue, covering bread (Contributions 1–5), biscuits (Contribution 6), and pasta (Contributions 7–8), point to a common conclusion: the quality of grain-based foods depends not only on the ingredients used, but also on how formulation and processing interact to shape structure, functionality, and acceptability.

This clearly emerges from studies on bread enriched with tiger nut flour (Contribution 3), bran from ancient grains (Contribution 2), vitamin D3 and fibre (Contribution 5), or sourdough from germinated seeds (Contribution 1), as well as from papers on pasta reformulated with malted triticale (Contribution 8) sorghum or cork oak flour (Contribution 7). Taken together, these contributions show that traditional cereal-based foods are very useful model systems for testing whether nutritional improvement or utilization of unconventional ingredients can be translated into products with suitable technological performance and acceptable sensory quality. In different ways, all these studies reflect the same effort: innovating well-known foods without losing the characteristics that make them successful and relevant in practice.

At the same time, the papers make clear that the use of alternative ingredients is never only a compositional issue. Ingredients such as tiger nut flour (Contribution 3), bran (Contribution 2), sprouted mung bean flour (Contribution 6), germinated lentils (Contribution 1), sorghum and cork oak flour (Contribution 7), or malted triticale (Contribution 8) alter the systems profoundly. They affect dough rheology, gas retention, crumb structure, cooking behaviour, texture, colour, and/or shelf-life. For this reason, one of the clearest messages of this Special Issue is that reformulation cannot be reduced to the simple addition of nutritionally attractive ingredients. The real question is not whether nutritional improvement is possible, but under which conditions it can be achieved without compromising the structural, technological, and sensory properties that define the product.

This leads to another important theme running through the Special Issue: process matters as much as ingredient choice [3]. Germination (Contributions 1 and 6) malting (Contribution 8), sourdough fermentation (Contribution 1), and dry fractionation (Contribution 9) are not secondary steps applied after formulation. They are enabling strategies that can change the nutritional and technological properties of the raw material itself. Their value lies in their ability to turn compositional potential into functional performance.

A further point raised by the collection of studies is that reformulation does not end with product development. After evaluating the nutritional quality of wholegrain cereal-based products sold on the Italian market [4], Martini et al. (Contribution 4) dealing with front-of-pack labelling and packaged bread broadens the discussion by showing that improvements in nutritional quality are not always captured equally well by existing labelling schemes. This is an important reminder that innovation in grain-based foods also depends on whether the wider food environment is able to recognize, communicate, and possibly reward these efforts.

Overall, the papers collected here suggest that food scientists can capitalize on the opportunities offered by combining the design of composition, structure, process, and purpose. Grain-based products still offer considerable room for improvement because the ways in which we formulate, process, and evaluate them are still evolving. Perhaps this is the most lasting message of the Special Issue: the future of grain-based foods will depend on our ability to connect technological knowledge with nutritional and social needs.

## Data Availability

The data described here are publicly available in the Special Issue “Traditional and Non-Traditional Grain-Based Products: Addressing Challenges Through Formulation and Processing”.
